# Resolution of Refractory Status Epilepticus With Ketamine Without Intubation in a Patient With Stroke-Like Migraine Attacks After Radiation Therapy (SMART) Syndrome

**DOI:** 10.1155/crnm/3203322

**Published:** 2025-02-04

**Authors:** Kyle N. Kaneko, Pablo Read, John M. Eaton, Yoshie Umemura, Justin L. Hoskin

**Affiliations:** ^1^Department of Neurology, Barrow Neurological Institute, Phoenix, Arizona, USA; ^2^Department of Neurology, Creighton University School of Medicine, Phoenix, Arizona, USA

**Keywords:** ketamine, oligodendroglioma, SMART syndrome, status epilepticus

## Abstract

Stroke-like migraine attacks after radiation therapy (SMART) syndrome is an infrequently reported complication arising years after radiation therapy that manifests as a reversible syndrome marked by migraine-like headaches, focal neurologic signs, and/or seizures. Refractory status epilepticus (RSE) associated with SMART syndrome is rare and can be challenging to treat. Valproic acid has been reported to improve seizures in RSE in SMART syndrome in a few case reports and may be ideal for SMART syndrome, given its use in the treatment of migraines and seizures. Ketamine has been used in RSE and a few instances in SMART syndrome. Here, we present a case of refractory focal status epilepticus in a patient with SMART syndrome who was treated with ketamine, which resolved seizures without the need for intubation.

## 1. Introduction

Stroke-like migraine attacks after radiation therapy (SMART) syndrome is a rare and delayed consequence of brain radiotherapy. This condition typically emerges years or even decades after radiation exposure, although instances within 2 years have occurred [1]. The disorder's etiology remains unclear, although it is thought to involve radiation-induced neurotoxicity leading to endothelial damage, vascular dysfunction, and failure of autoregulation with neuronal impairment [2, 3].

SMART syndrome has several findings that should raise suspicion for it. However, because of the syndrome's rarity and variable presentation, the diagnosis remains one of exclusion. It classically presents 2–10 years postcranial radiation therapy with recurrent headaches, seizure activity, and paroxysmal neurological deficits such as aphasia, hemianopsia, or neglect [4]. Other manifestations include visuospatial deficits, facial droop, and confusion [5]. In 2006, Black et al. proposed a formal diagnostic criterion for SMART syndrome that comprises of (1) a remote history of cerebral irradiation, (2) reversible, prolonged unilateral cortical symptoms beginning years after radiation therapy, (3) unilateral, transient, diffuse cortical gray matter enhancement sparing white matter, and (4) the exclusion of other potential underlying causes [5].

Gadolinium-enhanced brain magnetic resonance imaging (MRI) is the gold standard in evaluating SMART syndrome. MRI findings may include gyriform enhancement, cortical microhemorrhages, local linear hypointensity on susceptibility-weighted imaging (SWI) and T2, subcortical white matter edematous changes, locally increased cerebral blood volume, and low apparent diffusion coefficient. MRI findings are transient and are primarily seen in the parieto-occipital cortex with sparing of the white matter [5, 6]. Currently, no recommendations exist on the most effective treatment strategy for SMART. Some believe calcium channel blockers have a role as adjuvant drugs for headaches. Verapamil has well-documented success serving as an adjuvant and long-term prophylaxis, often paired with aspirin, which is thought to treat dysfunctional cerebral vasoreactivity caused by radiation [7].

Seizures are a common feature of SMART syndrome, with case series finding seizures in 35%–64% of patients [8, 9]. Although studies suggest seizures may be a hallmark feature of SMART syndrome, refractory status epilepticus (RSE) in SMART syndrome is extremely rare, with only a few cases reported [10–13]. All cases of RSE seen in SMART syndrome were given corticosteroids and required aggressive treatment with antiseizure medications (ASMs), with most requiring anesthesia consisting of ketamine and midazolam [13–15]. Here, we describe a case of focal RSE in a patient with SMART syndrome that resolved with ketamine infusion without the need for intubation.

## 2. Case Presentation

**Figure 1:**
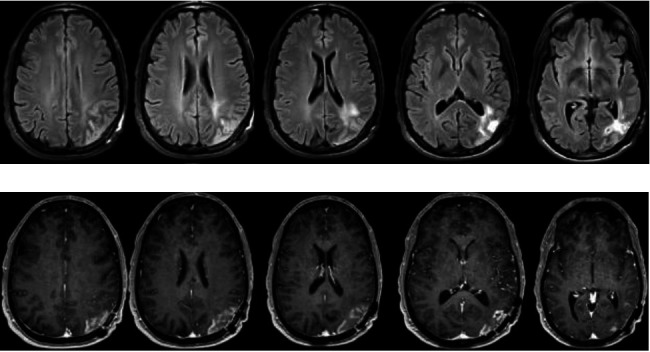
Brain magnetic resonance imaging with and without contrast. Brain MRI: (a) T2 FLAIR showing left parieto-occipital resection cavity with surrounding T2 FLAIR hyperintensity. (b) T1 postcontrast showing nonspecific faint rim of peripheral ribbon like enhancement.

**Figure 2:**
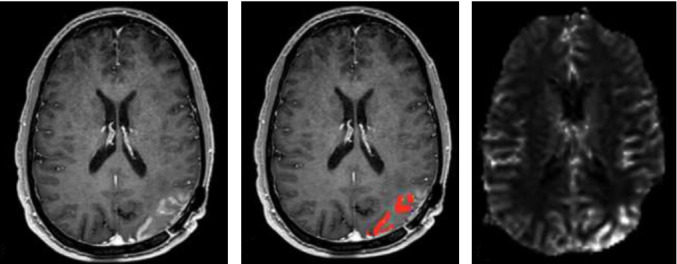
Brain magnetic resonance imaging perfusion (a) T1 postcontrast axial MRI showing nonspecific faint rim of peripheral ribbon like enhancement in the left parieto-occipital region. (b) MRI perfusion imaging showing increased cerebral blood flow in the left parieto-occipital region. (c) Increased cerebral blood volume in the left parieto-occipital region in a cortical pattern.

This is a 36-year-old man with a history of a left parietal oligodendroglioma World Health Organization grade 2 with confirmed isocitrate dehydrogenase 1 mutation and 1p19q codeletion. He initially presented with a generalized tonic-clonic seizure which led to the diagnosis. He underwent left parietal craniotomy for tumor resection and received upfront radiation therapy to total 50.4 gray in 28 fractions with concurrent temozolomide, followed by 12 cycles of adjuvant temozolomide. About 2.5 years after the brain tumor diagnosis and about 1 year after completion of radiotherapy, he presented with a five-month history of worsening headaches and right visual field blurriness and intermittent flashing lights. His headache onset coincided with self-discontinuation of levetiracetam 1000 mg twice a day due to having been asymptomatic for 2 years. He initially saw his primary care physician for the left-sided headache, rated at 8/10 at its worst, and was trialed on ubrogepant, acetaminophen, aspirin, caffeine, and rimegepant with no relief. His headaches were associated with flashing lights in both eyes in the middle of his visual field, lasting three to five minutes before disappearing, occurring almost every 20–30 min. On physical exam, his vital signs were normal. He was alert and oriented to person, place, time, and situation. Cranial nerves were intact aside from a right homonymous hemianopsia on visual fields. Motor and sensory exams were unremarkable. A brain MRI with and without contrast showed a left parieto-occipital resection cavity with a nonspecific faint rim of peripheral enhancement with enhancement having a gyriform pattern and associated T2 fluid-attenuated inversion recovery (FLAIR) hyperintensity (Figure 1). MRI perfusion imaging showed increased cerebral blood volume and blood flow, mainly in the periphery in a cortical pattern (Figure 2). Neuro-oncology and radiation oncology were consulted, and it was concluded that the MRI findings were unlikely to be a tumor progression but instead were most consistent with SMART syndrome given the presenting symptoms, history of radiation, and the imaging finding of ribbon-like gyriform.

**Figure 3:**
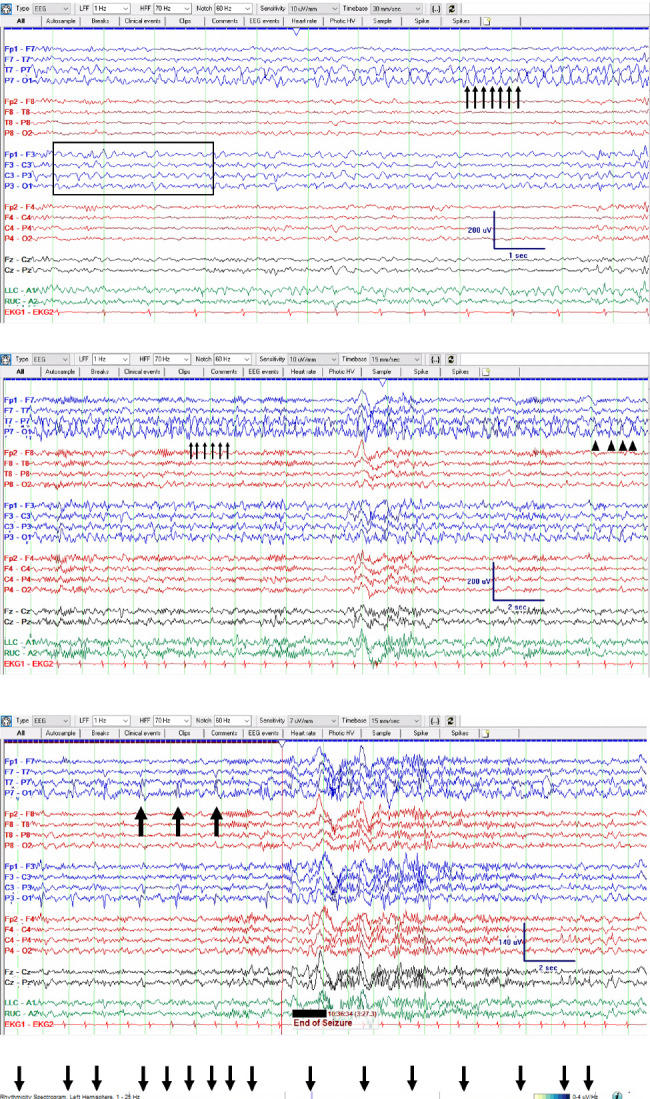
Initial electroencephalogram (EEG). (a) The start of a focal seizure. In the box shows left posterior temporal focal slowing (not an ictal pattern). This becomes sharper and quasirhythmic in the 3–6 Hz delta-theta range as the seizure starts, with phase reversal sharps (arrows). (b) Development of a clearer ictal pattern. Focal 4–5 Hz theta slowing spreads broadly over the left parasagittal chain (box). P7 sharps become more apiculate spikes with clear rhythmicity at 6 Hz (arrows). (c) Further ahead in the seizure and on a compressed time scale, left posterior ictal pattern slows from sharp quasirhythmic 3–4 Hz activity (arrows) to 2 Hz discharges (arrowheads). (d) End of seizure—black arrows indicate ongoing left posterior discharges slowing to every 1.5 s, then stopping and as the seizure ends. Total duration of this seizure is 3 min, 27 s. (e) Quantitative EEG trends showing a period of 4 h shortly after the EEG is first connected, with the vertical blue line indicating the start of the index seizure shown. Rhythmicity spectrogram and FFT spectrogram on the left hemisphere, as well as asymmetry spectrogram and amplitude-integrated EEG show greater power (uV) over the left compared to the right. During this 4 h period, there were 16 focal left posterior temporal/occipital seizures.

The patient was started on continuous video electroencephalogram (EEG), which showed left parieto-occipital abundant interictal epileptiform discharges and focal left posterior quadrant seizures occurring at a rate of 3-6 per hour (Figure 3). The average seizure duration was about 2–5 min, with the patient meeting criteria for nonconvulsive status epilepticus. He was loaded with 4.5 g of levetiracetam but continued to have focal seizures. He was then loaded with valproic acid (VPA) 3 g and started on maintenance of 500 mg three times a day. He continued to have focal seizures seen on EEG despite his reported improvement in his visual symptoms. Lacosamide 300 mg was loaded, but his EEG continued to demonstrate seizures at a similar rate of 3-6 per hour, so clobazam 10 mg twice a day was started. At that time, he was on four maintenance ASMs without any improvement in seizure frequency as confirmed by EEG. In light of case reports of SMART syndrome with benefits from 1 g of intravenous methylprednisolone, three doses of 1 g of methylprednisolone were administered, unfortunately leading to no improvement on EEG. He was then given zonisamide with no improvement in his EEG.

**Figure 4:**
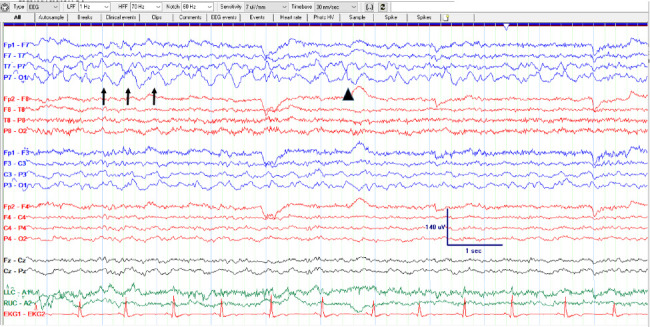
EEG after ketamine infusion. The end of a later seizure after ketamine is started. As the seizure resolves, the ictal pattern is replaced by left posterior delta activity (arrows) and sharps (arrowhead) which improves postictally.

**Figure 5:**
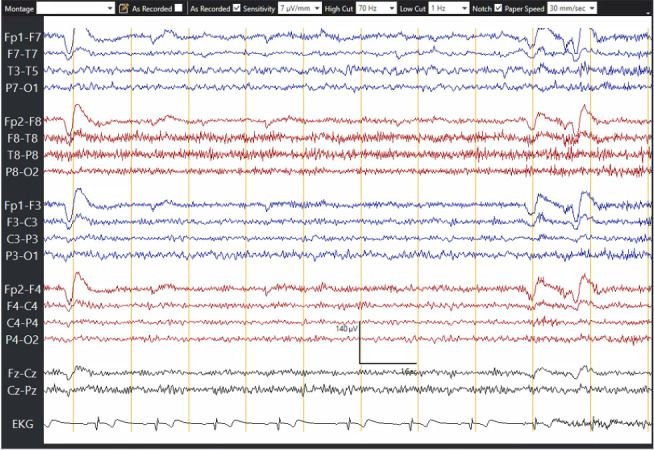
72 h ambulatory EEG after discharge. Frequent focal slowing in the left posterior region without epileptiform discharges or seizures.

At that point, he was in nonconvulsive status epilepticus for 3 days and was transferred to the intensive care unit for ketamine infusion. The decision was made not to try aggressive anesthetic treatments and intubate with ventilatory support as his seizures did not have any observable clinical manifestations and did not manifest as secondary bilateral tonic-clonic seizures. The goal of ketamine was to titrate slowly to reduce seizures while avoiding intubation if possible. Ketamine was administered with an 86 mg bolus (1 mg/kg) and maintenance rate was placed at 0.5 mg/kg/min with a max infusion rate of 2.58 mL/hr. After he received the ketamine bolus and maintenance infusion, his seizure frequency went from 3-6 an hour to 2-4 an hour. The next day, his seizure frequency decreased further to 0-2 an hour, and eventually, he had no seizures at the end of the second day (Figure 4). He was on the ketamine drip for 2 days with no increase in infusion rate. During this time, the patient remained alert and able to follow commands but did endorse significant anxiety and mood changes, with outbursts of crying at times. He also admitted to auditory and visual hallucinations at times. After ketamine was discontinued, his mood symptoms and hallucinations resolved. He was discharged home with five ASMs and was seen in the clinic three weeks later for a 72-h EEG. He stopped taking zonisamide after being discharged due to decreased appetite and significant nausea. His repeat 72-h EEG showed no seizures or epileptiform activity (Figure 5). He reported resolution of his headaches and episodic flashing lights. He was instructed to continue taking levetiracetam, VPA, lacosamide, and clobazam. Although a follow up MRI brain was not completed, the cortical pattern of increased cerebral blood flow was felt to be from treatment effect rather than tumor recurrence. A follow up MRI brain was not able to be obtained since the patient was lost to follow up.

## 3. Discussion

SMART syndrome is a rare and often reversible consequence of cranial irradiation. Given its variable presentation and ability to mimic and coincide with other common medical conditions, the diagnosis can be challenging to establish. Patients benefit from prompt recognition, and workup should include gadolinium-enhanced brain MRI. The hallmark of SMART syndrome is prominent and often unilateral gyral enhancement with mild mass effect and cortical thickening (hyperintense in FLAIR and T2) with or without diffusion restriction [1, 2]. Our patient reported worsening migraine-like headaches with associated right visual field blurriness and flashing lights following cessation of levetiracetam. New enhancement on brain MRI in the setting of new focal neurologic deficits initially raised concern for tumor recurrence. However, the symptoms, history, and imaging findings made SMART syndrome more likely. When reviewing imaging for possible tumor recurrence, it is important to consider tumor-like lesions in the differential. For patients who have previously been cured of cancer, learning about a possible recurrence can be psychologically devastating. Recognizing nonprogression etiologies in brain tumor patients is vital for planning treatment, estimating outcomes and quality of life, and improving psychological health in patients.

Our patient's EEG showed focal onset with retained awareness left posterior quadrant seizures occurring at a rate of 3–6 per hour but refractory to multiple ASMs. Pulse steroids were then trialed to avoid general anesthesia and intubation, with no improvement in the EEG, and further escalation of care was needed. Steroid pulse therapy, although not the standard of care, has been shown in cases to improve symptoms in SMART syndrome, although the role of steroids in the treatment of seizures in SMART syndrome is still unclear [15]. RSE in SMART syndrome is extremely rare, with only isolated cases reported [14]. Like our patient, other patients with RSE in SMART syndrome received pulsed steroid therapy along with multiple ASMs. Two patients had resolution of seizures with pulsed steroid therapy, VPA, and one additional ASM. However, one patient required general anesthesia with an anesthetic coma being induced [15]. VPA has been used in seizures in SMART syndrome in a few case reports, although VPA alone did not resolve the seizures. No definitive mechanism has been reported, and no recommendation on using VPA first line in SMART syndrome has been proposed. Given that VPA is used to treat both migraines and seizures, it theoretically is the ideal agent for seizures in SMART syndrome [16]. VPA's mechanism of action for seizures is suspected to be from sodium channel inactivation and inhibition of gamma-aminobutyric (GABA) transaminase, which increases levels and activity of GABA. This enhanced GABA is also thought to be a potential mechanism of action for preventing migraines. VPA also acts on the brain's serotonin system through GABA-mediated inhibition, raising the threshold of the onset of migraines. VPA is also proposed to reduce vasodilation, although the mechanism is unclear [17].

Ketamine has been shown in a few cases to resolve seizures in RSE in SMART syndrome, but all cases required intubation [12, 13]. As status epilepticus progresses, GABA receptors on the postsynaptic membrane decrease while glutamatergic N-methyl-D-aspartate (NMDA) receptors increase [18]. Ketamine is a noncompetitive antagonist of NMDA receptors and has been used to treat status epilepticus successfully [18]. Typical dosages for ketamine include a 1.5 mg/kg loading dose followed by an average infusion rate of 2.75 mg/kg/hour for 4 days [19]. One proposed mechanism for ketamine use in SMART syndrome is secondary to impaired neurons from radiation-induced mitochondrial damage leading to dysfunction in the sodium/potassium ATPase. Due to the impaired sodium/potassium ATPase, glutamate is shifted to the extracellular space, which binds to NMDA receptors, leading to a large influx of calcium ions, increasing neuronal hyperexcitability [20]. Theoretically, ketamine can block excess glutamate from binding NMDA receptors, reducing calcium influx and neuronal hyperexcitability. Our patient represents the first reported case of successful management of RSE in SMART syndrome using a ketamine bolus dose of 1 mg/kg followed by a maintenance dose of 0.5 mg/kg/hr, without requiring intubation or additional anesthetic agents. Further research is necessary to clarify ketamine's role in the pathophysiology of SMART syndrome.

## 4. Conclusion

This case demonstrates refractory focal status epilepticus in a patient with SMART syndrome and the use of ketamine to resolve seizures and avoid intubation. Ketamine can be considered as a potentially effective option in status epilepticus in the context of SMART syndrome that is refractory to pulsed steroid therapy and trials of ASMs. Further studies are needed to better understand the efficacy and safety of ketamine in managing seizures and status epilepticus in SMART syndrome.

## Data Availability

The data that support the findings of this study are available from the corresponding author upon reasonable request.

## References

[1] Winter S. F., Klein J. P., Vaios E. J. (2021). Clinical Presentation and Management of SMART Syndrome. *Neurology*.

[2] de Oliveira Franco Á, Anzolin E., Schneider Medeiros M., Machado Castilhos R., Targa Martins R., Moser Filho H. (2021). SMART Syndrome Identification and Successful Treatment. *Case Reports in Neurology*.

[3] Biju R. D., Dower A., Moon B. G., Gan P. (2020). SMART (Stroke-like Migraine Attacks after Radiation Therapy) Syndrome: A Case Study with Imaging Supporting the Theory of Vascular Dysfunction. *Am J Case Rep*.

[4] Bompaire F., Zinchenko L., Lahutte M. (2017). SMART Syndrome: Classic Transient Symptoms Leading to an Unusual Unfavorable Outcome. *Revue Neurologique*.

[5] Black D. F., Bartleson J. D., Bell M. L., Lachance D. H. (2006). SMART: Stroke-like Migraine Attacks after Radiation Therapy. *Cephalalgia*.

[6] Ota Y., Leung D., Lin E. (2022). Prognostic Factors of Stroke-like Migraine Attacks after Radiation Therapy (SMART) Syndrome. *AJNR Am J Neuroradiol*.

[7] Kerklaan J. P., Lycklama á Nijeholt G. J., Wiggenraad R. G., Berghuis B., Postma T. J., Taphoorn M. J. (2011). SMART Syndrome: a Late Reversible Complication after Radiation Therapy for Brain Tumours. *Journal of Neurology*.

[8] Di Stefano A. L., Berzero G., Ducray F. (2019). Stroke-like Events after Brain Radiotherapy: a Large Series with Long-Term Follow-Up. *European Journal of Neurology*.

[9] Rigamonti A., Lauria G., Mantero V., Filizzolo M., Salmaggi A. (2016). SMART (Stroke-like Migraine Attack after Radiation Therapy) Syndrome: a Case Report with Review of the Literature. *Neurological Sciences*.

[10] Ferlazzo E., Ascoli M., Gasparini S. (2018). Seizures with Migraine-like Attacks after Radiation Therapy (SMART): A New Meaning of an Old Acronym. *Seizure*.

[11] Ascoli M., Ferlazzo E., Cianci V., Aguglia U., Gasparini S. (2021). SMART: Stroke-like Migraine Attacks after Radiation Therapy or Seizures with Migraine-like Attacks after Radiation Therapy? Terms Do Matter in Clinical Practice. *Neurological Sciences*.

[12] Panigrahy N., Aedma S., Lee M. (2022). Stroke-Like Migraine Attacks after Radiation Therapy (SMART) Syndrome Presenting with Recurrent Seizures: A Case Study. *Cureus*.

[13] Hametner E., Unterberger I., Lutterotti A. (2015). Non-convulsive Status Epilepticus with Negative Phenomena--a SMART Syndrome Variant. *Seizure*.

[14] Jaraba S., Puig O., Miró J. (2015). Refractory Status Epilepticus Due to SMART Syndrome. *Epilepsy and Behavior*.

[15] Jia W., Saito R., Kanamori M., Iwabuchi N., Iwasaki M., Tominaga T. (2018). SMART (Stroke-like Migraine Attacks after Radiation Therapy) Syndrome Responded to Steroid Pulse Therapy: Report of a Case and Review of the Literature. *eNeurologicalSci*.

[16] Mathew N. T., Saper J. R., Silberstein S. D. (1995). Migraine Prophylaxis with Divalproex. *Archives of Neurology*.

[17] Pini L. A., Lupo L. (2001). Anti-epileptic Drugs in the Preventive Treatment of Migraine Headache: a Brief Review. *The Journal of Headache and Pain*.

[18] Fang Y., Wang X. (2015). Ketamine for the Treatment of Refractory Status Epilepticus. *Seizure*.

[19] Gaspard N., Foreman B., Judd L. M. (2013). Intravenous Ketamine for the Treatment of Refractory Status Epilepticus: A Retrospective Multicenter Study. *Epilepsia*.

[20] Ota Y., Liao E., Shah G., Srinivasan A., Capizzano A. A. (2023). Comprehensive Update and Review of Clinical and Imaging Features of SMART Syndrome. *AJNR Am J Neuroradiol*.

